# Wavelet Entropy: A New Tool for Edge Detection of Potential Field Data

**DOI:** 10.3390/e25020240

**Published:** 2023-01-28

**Authors:** Divyanshu Dwivedi, Ashutosh Chamoli, Sandip Kumar Rana

**Affiliations:** 1 CSIR-National Geophysical Research Institute, Hyderabad 500007, India; 2 Department of Earth Sciences, Indian Institute of Technology Roorkee, Roorkee 247667, India

**Keywords:** wavelet space entropy, edge detection, potential field, Bishop model, Delhi fold belt

## Abstract

Subsurface source boundary identification is a major step in the interpretation of potential field anomalies in geophysical exploration. We investigated the behavior of wavelet space entropy over the boundaries of 2D potential field source edges. We tested the robustness of the method for complex source geometries with distinct source parameters of prismatic bodies. We further validated the behavior with two datasets by delineating the edges of (i) the magnetic anomalies due to the popular Bishop model and (ii) the gravity anomalies of the Delhi fold belt region, India. The results showed prominent signatures for the geological boundaries. Our findings indicate sharp changes in the wavelet space entropy values corresponding to the source edges. The effectiveness of wavelet space entropy was compared with the established edge detection techniques. The findings can help with a variety of geophysical source characterization problems.

## 1. Introduction 

Source edges have a key role in modeling and interpreting potential field data. They are used to determine the boundaries of geological formations in the form of faults and contacts [1,2,3]. It is possible to create an edge-location map with minimal computational effort and without any prior geological expertise. The amplitude of gravity and magnetic anomalies show changes due to the different geometries and property contrasts of complex subsurface sources. Horizontal derivatives, analytical signals, tilt angles, theta maps, and image processing techniques are common source edge identification techniques. Source edge detection methods have utilized vertical derivatives [4,5]. The horizontal derivative approaches further locates the horizontal source positions [6,7]. In the total horizontal derivative (THDR) method (Equation (1)), the edges of the anomaly sources are determined by the highest amplitude values. The square root of the sum of the squares of the directional derivatives of the potential field data is used to define the analytical signal amplitude (ASA) (Equation (2)). The edges of the source are represented by the maxima values. When noise, poor data quality, or anomalies resulting from shallow and deep sources are present, the derivative filters’ drawbacks become apparent [8]. Phase-based filters such as tilt angle (TDR, Equation (3)), which are computed by normalizing the vertical derivative with respect to the total horizontal derivative, have been developed as solutions for source edge detection [9,10,11]. Low-amplitude anomalies and high-level noise in the data are shortcomings of the tilt approach [12]. In the presence of deep sources, the tilt filter is recommended rather than the derivative filters [8]. The theta map [13] is a phase-based filter that is evaluated as the total horizontal derivative normalized by the analytical signal amplitude (Equation (4)). Although the analytical signal amplitude improves the smaller amplitude anomalies, it displays poor resolution for deep sources and noise [5]. An edge detection method was also proposed based on derivatives and tilt angle concepts [14]. Gradient and arcsin functions are also combined and used to determine edges [15]. Due to interference effects, these techniques have limitations in the presence of nearby vertically shallow and deep sources, and the separation of sources becomes difficult.

(1) $$THDR=\sqrt{{\left(\frac{\partial f}{\partial x}\right)}^{2}+{\left(\frac{\partial f}{\partial y}\right)}^{2}}$$

where f is the gravity or magnetic anomaly, and $\frac{\partial f}{\partial x}$ and $\frac{\partial f}{\partial y}$ are the data gradients in the x and y directions.

(2) $$ASA=\sqrt{{\left(\frac{\partial f}{\partial x}\right)}^{2}+{\left(\frac{\partial f}{\partial y}\right)}^{2}+{\left(\frac{\partial f}{\partial z}\right)}^{2}}$$

where $\frac{\partial f}{\partial z}$ is the data gradient in the z direction.

(3) TDR=tan−1(∂f∂zTHDR)

(4) $$THETA=\cos^{-1}\left(\frac{THDR}{ASA}\right)$$

Wavelet methods have shown significant contributions in different fields: gravity and magnetic source characterization and source edge detection [3,16,17,18], climatic signals [19], tsunami warning [20,21], and different time series analyses [22,23]. Shannon entropy is a measure of information of any distribution and has been found to be helpful for tsunami warnings [24,25], climatology and hydrology [26,27], and earthquake analysis [28,29] in past case studies. Wavelet-based entropy has an advantage over Fourier-based approaches in handling the nonstationary behavior of datasets and is used for distinguishing the critical behavior in physical phenomena.

Wavelet entropy can be calculated either as wavelet time entropy for a 1D time signal [30] or as wavelet space entropy (WSE) for 2D spatial signals [31]. WSE is the extension of wavelet time entropy in 2D space, which uses wavelets to identify sudden changes in signals. Gravity and magnetic datasets are 2D spatial datasets, and thus wavelet space entropy can give useful information associated with the spatial changes related to subsurface sources. We investigated the behavior of WSE for the identification of edges of sources in 2D data of potential fields. We used wavelet decomposition to calculate the wavelet space entropy. We tested the method on synthetic magnetic datasets of complex geometries of sources with the addition of noise. We evaluate the performance of WSE in comparison to other established methods and the addition of noise. We further validated the behavior using realistic datasets of (i) magnetic anomalies of the Bishop model and (ii) gravity anomalies of the Delhi fold belt (DFB). We characterized the source edges in terms of the order/disorder behavior associated with multiwavelength subsurface sources. This method showed its usefulness in the delineation of source boundaries.

## 2. Methods

### 2.1. Wavelet Decomposition

We used a multiresolution wavelet analysis to decompose the two-dimensional potential field dataset. Mallat’s multiresolution analysis [32] decomposed the 2D dataset into low-frequency approximation coefficients and high-frequency detail coefficients with a scale-dependent hierarchy. The mathematical concepts of multiresolution decomposition were described in a previous work [3]. The wavelet transform (discrete or continuous) of a function is the improved version of the Fourier transform, and it provides frequency and time information simultaneously. In this case, we took Daubechies wavelet of order one (db1), which was found to be useful for edge detection. The db1 is one of the members of the orthogonal wavelet family, and associated filters have a nonlinear phase. The mathematical concepts of multiresolution wavelet decomposition are explained in detail in Appendix A (Equations (A1)–(A10)).

### 2.2. Wavelet Energy

The wavelet energy (WE) for decomposition level k can be given by [28]:

(5) $$WE_{k}\left(m,\;n\right)={\left|W_{\psi }^{H,V,D}\right|}^{2}$$

where $W_{\psi }^{H,V,D}$ represents the wavelet detail coefficients (horizontal, vertical, and diagonal) and (m, n) are the indices of the window (s, t). 

The mean wavelet energy (MWE) of the level k for the window (s, t) of length (L_x_, L_y_) can be given as:

(6) $$MWE_{k}^{\left(s,\;t\right)}=\frac{1}{N}\sum_{m=\left(s-1\right).L_{x}+1\;\;}^{s.L_{x}}\sum_{n=\left(t-1\right).L_{y}+1}^{t.L_{y}}WE_{k}\left(m,\;n\right)$$

where s varies from 1 to (p/L_x_), t varies from 1 to (q/L_y_), p and q are the sizes of the detail coefficient matrices, and N represents the total number of wavelet coefficients in level k. The mean wavelet energy was computed for all windows at a specific level of k using Equation (6). We tested different window sizes to calculate the WSE and found that it gave a good resolution of the sources with a window length of 1 × 1 at the first level. The resolution decreased with more decomposition levels due to the variable windowing effect. 

The total wavelet energy (TWE) for different decomposition levels (k = 1 to D) can be defined as:

(7) $$E_{tot}^{\left(s,t\right)}=\sum_{k=1}^{D}MWE_{k}^{\left(s,\;t\right)}$$

The relative wavelet energy (RWE) of the window (s, t) at decomposition level k can be defined as:

(8) $$RWE_{k}^{\left(s,\;t\right)}=\frac{MWE_{k}^{\left(s,\;t\right)}}{E_{tot}^{\left(s,t\right)}}$$

### 2.3. Wavelet Space Entropy

Shannon [33] applied the entropy concept in the information theory. Shannon entropy calculates the expected value of the information generated from a random variable. The concept was further extended for two dimensional cases using a directional Morlet wavelet to calculate wavelet space entropy [28] to detect different spatial complexities in earthquake analysis. The definition of entropy can also be understood in other types of distributions such as energy distribution based on wavelet coefficients [34,35]. Entropy-based edge detection without using wavelet decomposition coefficients is unsuitable due to its statistical properties and does not take into account the spatial properties of the pixel within the image [36]. Wavelet time entropy can be calculated by the multiresolution decomposition of a one-dimensional discrete time signal and then choosing a time window of a specific length to compute the average wavelet energy and relative wavelet energy to achieve the wavelet time entropy. In general, gravity and magnetic anomalies are 2D spatial datasets, and thus we utilized wavelet space entropy for edge detection. Our approach used wavelet decomposition and Shannon entropy together to determine the edges of the sources in 2D potential field data. The 2D WSE for the D level can be defined as [36]:

(9) $$S_{WT}^{\left(s,t\right)}=-\sum_{k=1}^{D}RWE_{k}^{\left(s,\;t\right)}*ln\left[RWE_{k}^{\left(s,\;t\right)}\right]$$

The calculation includes the following steps:

1. First, 2D potential field data with dimensions of M × N were decomposed into different wavelet decomposition levels (k = 1 to D) to obtain approximation coefficients $\;(W_{\phi }\left(j_{0},p,q\right)),\;$ and detail (horizontal, vertical, and diagonal) coefficients $(W_{\psi }^{H,V,D}\left(j,p,q\right))\;\;$ using Equations (A1)–(A10). The analyzing wavelet used was Daubechies of order one (db1) at the first level.
2. The horizontal and vertical detail coefficients $\left(W_{\psi }^{H}\left(j,p,q\right)\;\;\mathrm{and}\;W_{\psi }^{V}\left(j,p,q\right)\right)$ were combined and normalized to calculate the mean, total, and relative wavelet energies using a window of length L_x_ × L_y_ at a specific level using Equations (6)–(8). We avoided the utility of the diagonal detail coefficients $\left(W_{\psi }^{D}\left(j,p,q\right)\right)$ due to their poor resolution and noisy information.
3. The normalized wavelet space entropy was calculated with the relative wavelet energy using Equation (9).

The methodology was tested on synthetic as well as real cases, where synthetic cases represented the surface gravity or the magnetic response of the subsurface prismatic geometries similar to realistic subsurface source conditions and the real cases represented datasets acquired on the Earth’s surface that were interpreted to derive the subsurface geological structures. 

## 3. Synthetic Cases

Gravity and magnetic anomalies are responses of subsurface three-dimensional models at the surface. Such models are represented in terms of 3D geometry (length, width, and depth) and physical property contrasts, such as density or magnetic susceptibility for gravity or magnetic cases. Surface gravity or magnetic datasets are displayed in two dimensions as isocontour anomaly maps. In gravity and magnetic applications, three-dimensional subsurface bodies can be modeled using a collection of rectangular prisms [37]. 

**Figure 1:**
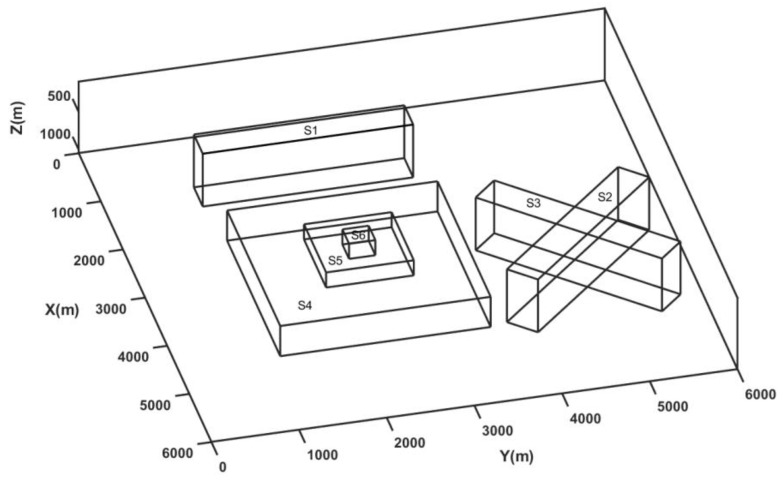
Three-dimensional synthetic model (M1) with six prisms.

**Figure 2:**
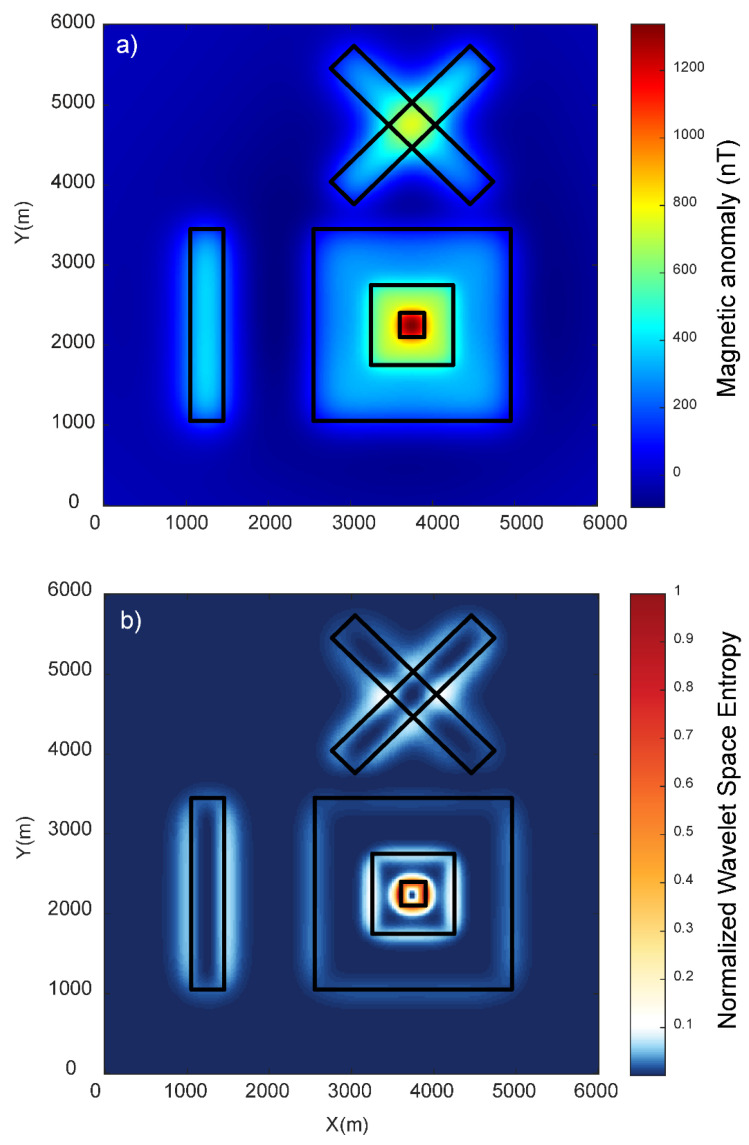
(**a**) Total magnetic anomaly generated due to 3D synthetic model (M1). The black rectangular lines show the actual upper edges of the prismatic sources. (**b**) Variations in the normalized wavelet space entropy of the magnetic anomaly. Note the sharp changes over the source edges.

Edge detection methods find limitations in resolving the edges for different scenarios such as the presence of different sizes of bodies, relatively shallow and deep sources, and the interference of nearby source anomalies. We generated a synthetic case of prismatic sources with different dimensions lying at various depths (model M1), which is summarized in Table 1 and shown in Figure 1, which solely mimicked all the above-mentioned critical scenarios. The dataset was produced on a 6000 × 6000 m grid with a spacing of 20 m for the 3D magnetic model. In model M1, different sizes of bodies were set up using prisms S3, S4, and S5; relatively deep and shallow sources were produced by prisms S2 and S6; and the interference of nearby sources was simulated by prisms S2, S3, S4, S5, and S6. Prism S1 synthesized the condition of an isolated deep source. The prismatic sources induced magnetization with the inclination and declination of 90°and 0° and remanent magnetization of 50°and 20°. We calculated the magnetic anomaly due to the complex sources of six prismatic magnetic bodies (S1, S2, S3, S4, S5, and S6) lying at different depths (Figure 2). This example checked the validation of the method for real scenarios with source depth sensitivity and interference effects in the total magnetic anomalies generated due to different sources (Figure 2a). The wavelet approximation and detail coefficients were calculated at the first level using the db1 wavelet with a window size of 1 × 1. The wavelet horizontal and vertical detail coefficients were combined to calculate the energies. We calculated the wavelet space entropy using the relative wavelet energy, which identified the upper edges of the isolated prism (S1) and interfering prisms (S2, S3, S4, S5, and S6) lying at different depths. Figure 2b shows the WSE plot superimposed over the upper surface of the prismatic source boundaries marked as black lines. The entropy showed sharp changes in the signal where the edge of the prismatic source lied. The source boundaries were discernable with relatively high entropy values, similar to the disordered behavior in the systems. The results indicated the effectiveness of wavelet space entropy analysis in resolving the boundaries of subsurface sources of varied dimensions and complex orientations. 

### 3.1. Performance Analysis

We tested the robustness of the signatures of the wavelet space entropy for edge detections by comparing it with established edge detection techniques, changing the geometries and orientations of the sources, and adding noise. 

#### 3.1.1. Comparison with Conventional Methods 

**Figure 3:**
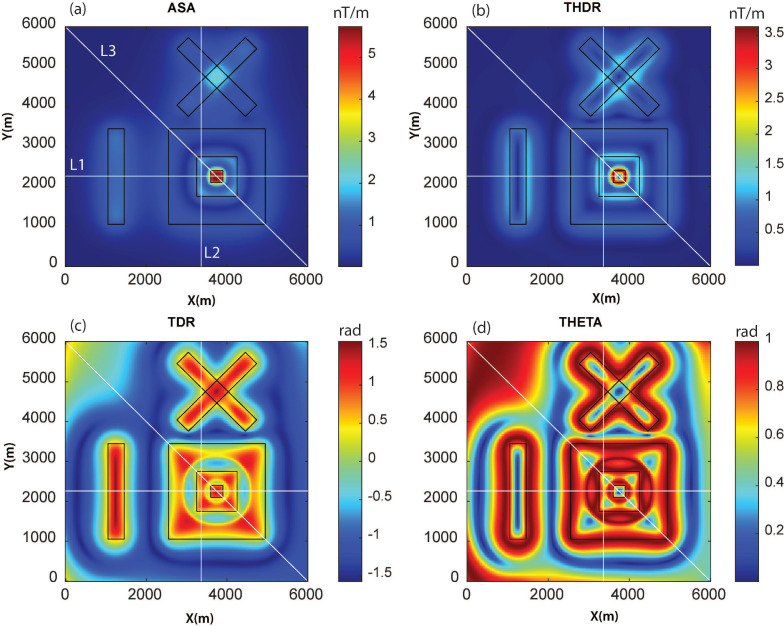
Comparison of different techniques to identify source edges for model M1. (**a**) ASA, (**b**) THDR, (**c**) TDR, (**d**) THETA. Three profiles (L1, L2, and L3) were used for error analysis (Figure 4).

**Figure 4:**
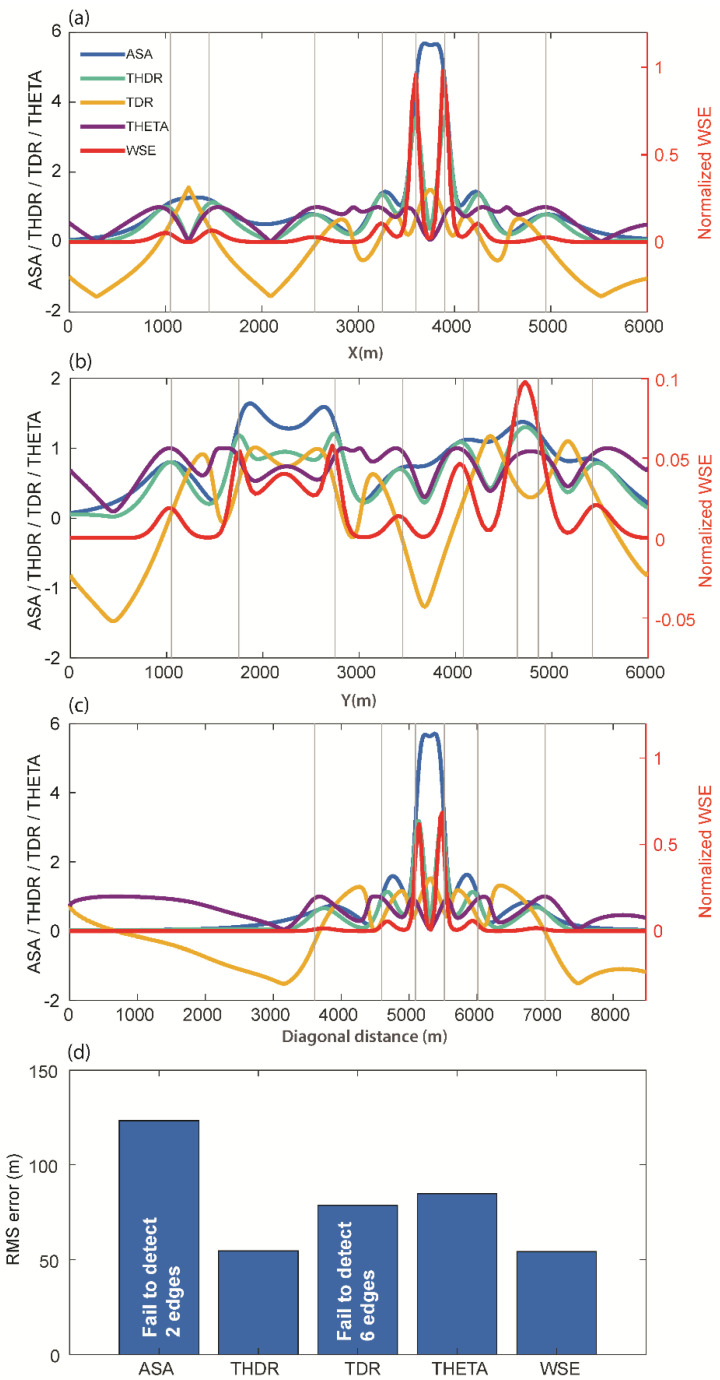
Comparison of the techniques for profiles (**a**) L1, (**b**) L2, and (**c**) L3 for model M1 and (**d**) the RMS errors for the different methods. In profile L2, over the two close edges of prisms S1 and S2, all the methods failed to resolve these due to high interference effects. Vertical grey lines indicate the actual source boundaries.

We examined the effectiveness of the WSE in comparison with established edge detection techniques (ASA, THDR, TDR, and THETA) for the magnetic anomaly of model M1 (Figure 3). We chose an approach similar to past studies and compared the methods first with a visual inspection, as practiced in geological interpretations. To quantitatively assess the performance, we calculated root-mean-square errors along selected profiles across main source boundaries. Among these methods, THDR and WSE produced clear edges for interpretation. We observed that the ASA method was not effective for deep sources such as S4 and S2. For the TDR and Theta map techniques, the edges deviated from the synthetic sources (Figure 3). In Figure 4, the actual edges (vertical grey lines) along the three profiles (L1, L2, and L3) are compared with the considered edge detectors’ signatures. This comparison shows how well THDR and WSE identified edges in comparison to the other methods. We observed that there was a shift in the signatures of the oblique prisms S2 and S3 in all methods, which was likely due to row/column-wise calculations in the algorithm. The RMS errors (Figure 4d) showed that WSE and THDR outperformed the performance analysis. In profile L2, over the two close edges of prisms S2 and S3, all the methods failed to resolve due to high interference effects. Because THDR and WSE performed equally, we restricted our further testing to these two methods.

**Figure 5:**
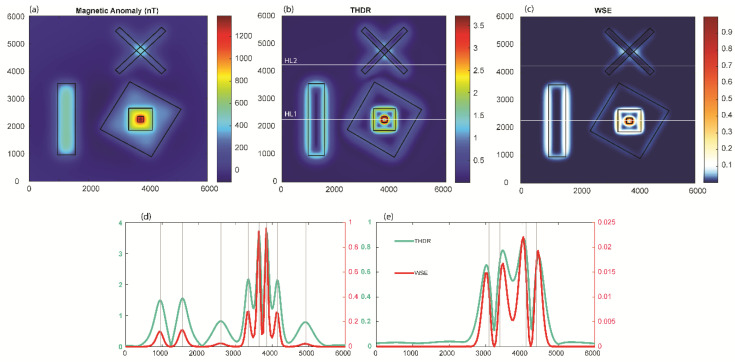
(**a**) Total magnetic anomaly generated due to 3D synthetic model (M2), (**b**) THDR, and (**c**) WSE maps. Comparison of THDR and WSE along the profiles of (**d**) HL1 and (**e**) HL2. The RMS errors for THDR and WSE were 44.6 and 44.2. Vertical grey lines indicate the actual source boundaries.

We further extended our analysis by varying the source parameters of model M1 to generate model M2 to test the robustness of the method. We introduced variations in the magnetic susceptibilities (positive and negative), total magnetization, and geometries due to different prisms to mimic different geological scenarios. The magnetic anomaly of model M2 is shown in Figure 5a, whereas the parameters of the source geometries are given in Table 2. The THDR and WSE plots of these prismatic sources are compared in Figure 5. Similarly, as in the previous case, THDR and WSE identified the source boundaries and matched the upper surfaces of the prismatic source boundaries (grey lines). Again, sources S2 and S3 exhibited a shift in the signature (Figure 5d,e). The RMS errors for THDR and WSE were 44.6 and 44.2, showing the better performances of these methods, even after changing the geometries and properties of the model. 

#### 3.1.2. Effect of Noise

**Figure 6:**
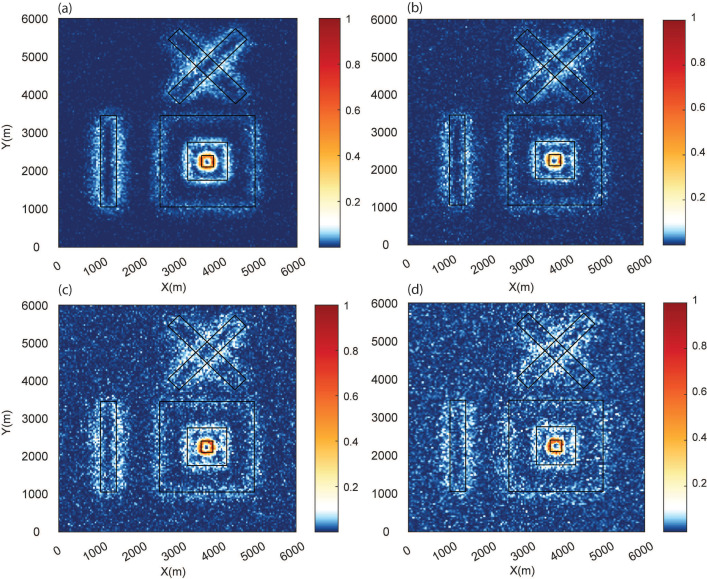
Normalized WSE after the addition of Gaussian noise with a mean of zero and standard deviations of (**a**) 5 nT, (**b**) 7 nT, (**c**) 8 nT, and (**d**) 10 nT to model M1.

**Figure 7:**
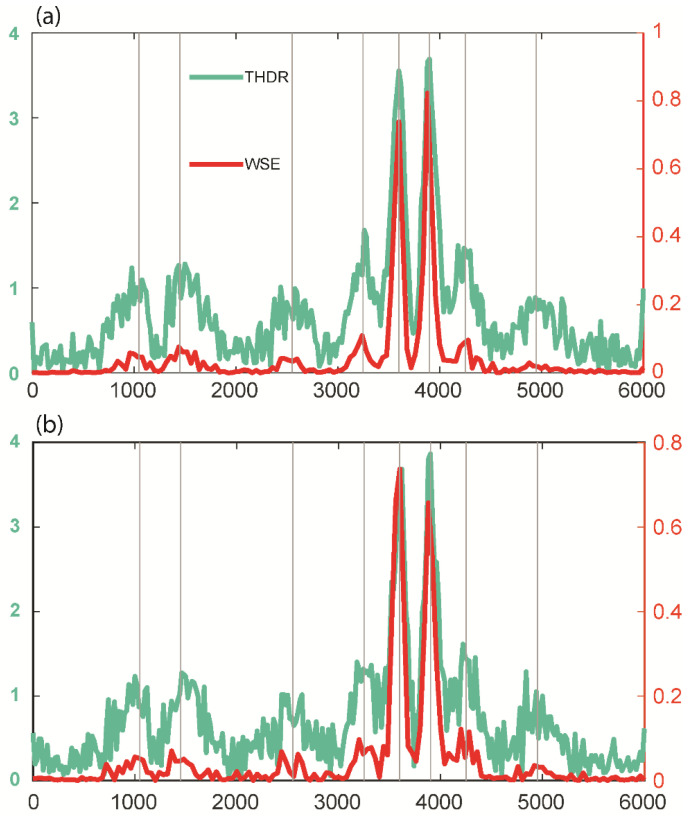
Comparison of THDR and WSE along profile L1 (Figure 6) for prismatic model M1 with Gaussian noise with standard deviations of (**a**) 5 nT and (**b**) 6 nT. The RMS errors for THDR and WSE were 55.4 and 49.2. Vertical grey lines indicate the actual source boundaries.

In general, noise from a variety of sources affects measured potential field data. We extended the testing by adding Gaussian noise to the total magnetic anomaly resulting from 3D synthetic model M1, as in many previous edge detection investigations [5,8], using Gaussian noise with a mean of zero and a standard deviation from 1 nT to 10 nT at the unit interval. Figure 6 shows the calculated normalized WSE for four cases with marginal to high noise (standard deviations of 5 nT, 7 nT, 8 nT, and 10 nT). For the noise with a standard deviation of 5 nT, the method was able to identify the edges, which were poorly resolved for noise with higher standard deviations. However, it was still possible to see the source edges in these situations. We also evaluated the effectiveness of THDR and WSE for the marginal cases of noise with standard deviations of 5 nT and 6 nT (Figure 7a), which showed the deterioration of peaks for the 6 nT cases. The WSE peaks were cleaner than the THDR peaks. The variations in THDR and WSE for the first case (Gaussian noise with standard deviation of 5 nT) along profile L1 demonstrated that both methods could locate the source edges, with RMS errors of 55.4 (THDR) and 49.2 (WSE). Since the peaks were not distinguishable for the second case, it was difficult to estimate the error. Our investigation demonstrated that THDR and WSE operated satisfactorily up to the Gaussian noise with a standard deviation of 5 nT and that the edges’ fingerprints for scenarios with rising standard deviations were still evident and could be helpful for source characterization. 

## 4. Application to Bishop Model

**Figure 8:**
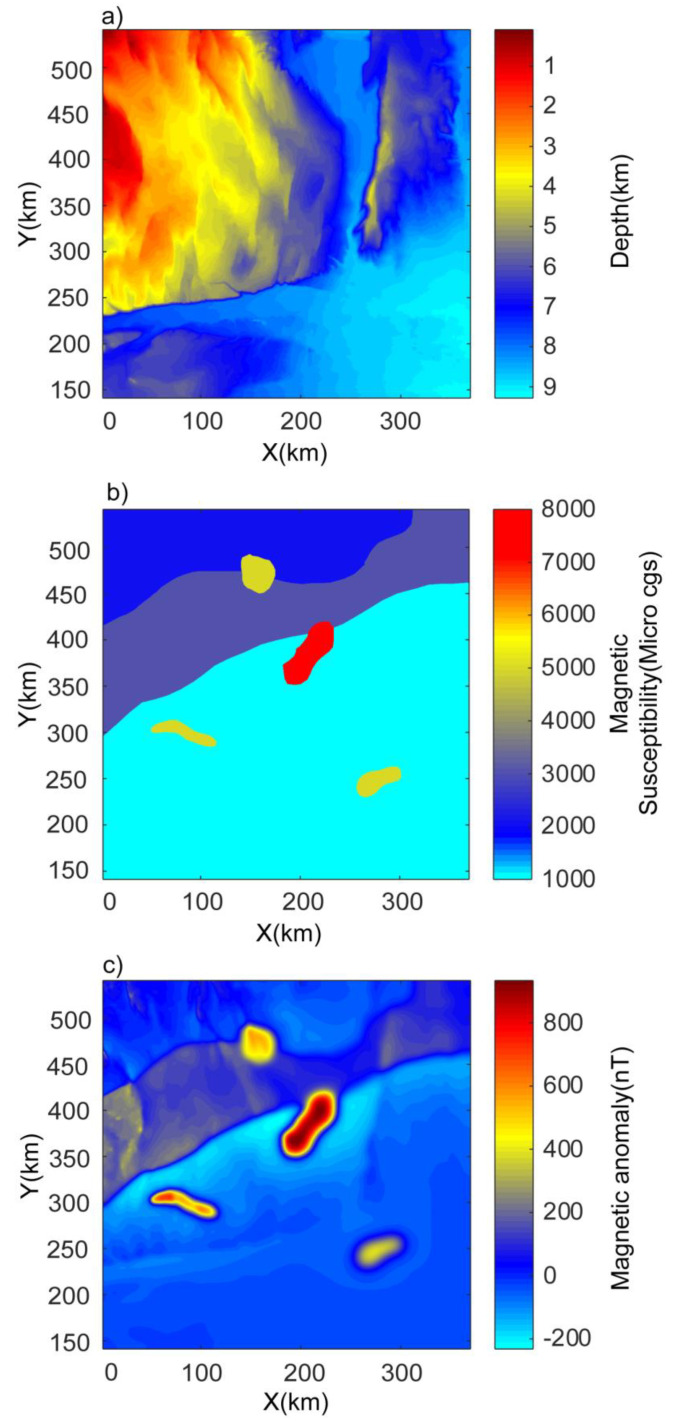
Bishop model. (**a**) Variations in depth of basement, (**b**) magnetic susceptibility variations in basement, and (**c**) total magnetic anomaly (after [3]).

**Figure 9:**
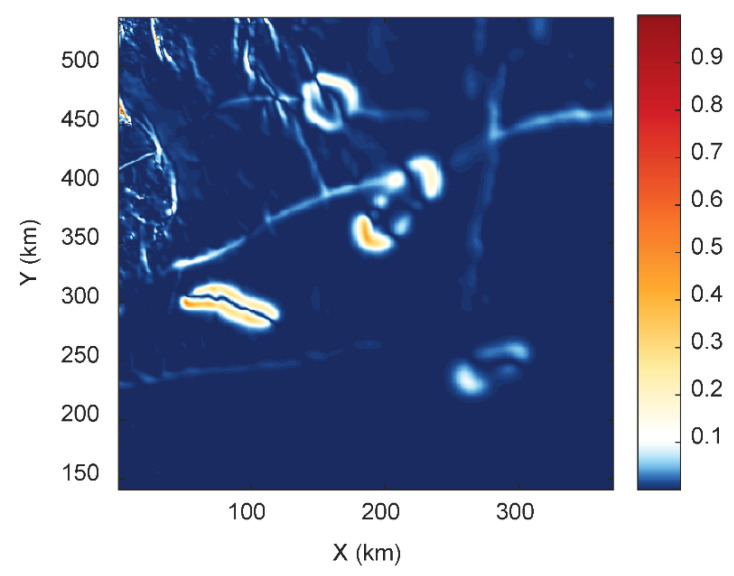
The edges identified by normalized wavelet space entropy for the magnetic anomaly of the Bishop model (Figure 8c).

The Bishop model is a 3D basement model that has been widely used to test different source edge detection methods in the past [3,38,39]. The Bishop model is a simulated basement relief model that was generated from the digital elevation model (DEM) of the Volcanic Tablelands close to Bishop, California. The DEM was scaled and shifted to make it comparable to a basin-scale magnetic basement surface [40]. Figure 8 shows the details of the Bishop model. The basement depth variations show the structural variations in the sources. There are magnetic susceptibility contrasts along two faults striking along the EW direction, a smaller-scale fault striking along the NS direction, and four isolated sources. The total magnetic anomaly reflects a realistic complex geological setting due to the presence of these sources. The WSE method was applied to the total magnetic anomaly (Figure 8c) of the Bishop model. We calculated the wavelet space entropy of the RWE, which showed sharp changes corresponding to these structures (Figure 9). The WSE clearly demarcated the source boundary of the EW- and NS-trending faults. The prominent signatures in WSE also confirmed the presence of the four isolated sources. The clustering of the points in the NW showed the signatures of the magnetic sources due to basement depth variations. The identified edges of the 3D isolated sources and the EW- and NS-trending fault structures were well correlated with Figure 8 as well as with the previous study [3]. The results imply that WSE is applicable for the sources due to complex subsurface geometries and property variations. 

## 5. Source Boundaries in the Delhi Fold Belt Region

**Figure 10:**
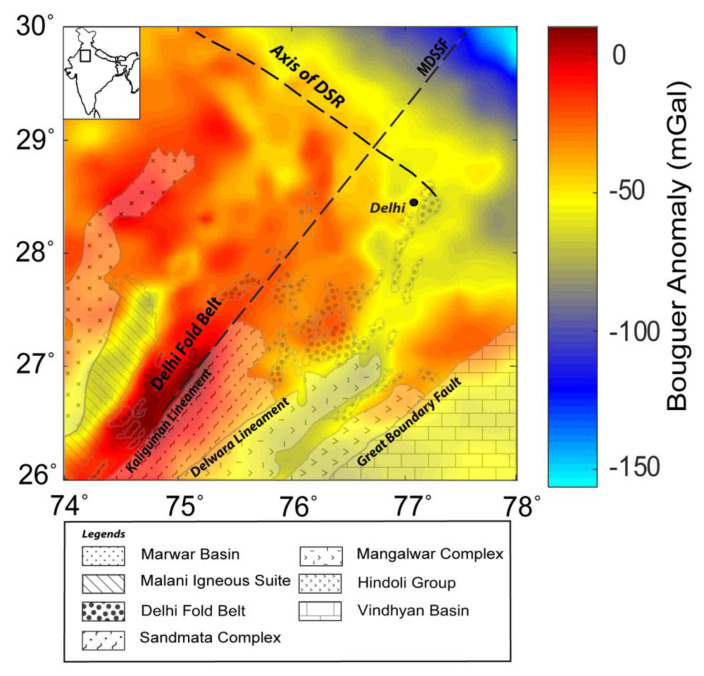
Geological map and the gravity anomaly of the Delhi fold belt and surrounding region (after [3,42]). Note the geological structures: the Delhi fold belt (DFB), the axis of the Delhi–Sargodha Ridge (DSR), the Mahendragarh–Dehradun Subsurface fault (MDSSF), the Great Boundary Fault (GBF), and the Kaliguman and Delwara lineaments.

**Figure 11:**
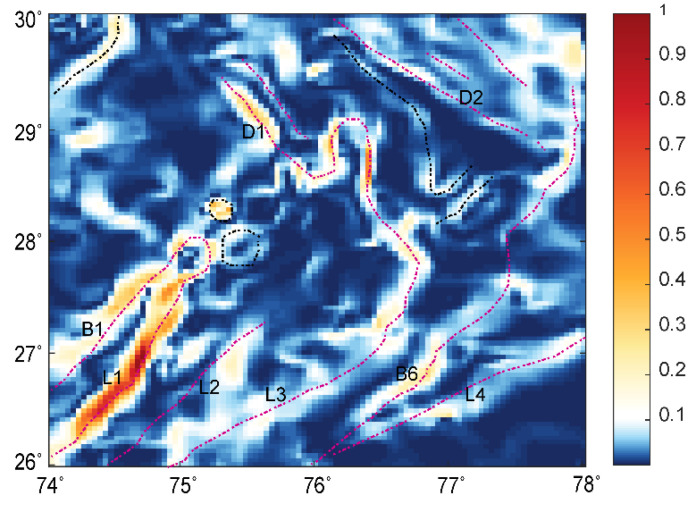
The edges identified using normalized wavelet space entropy of the Bouguer anomaly of the Delhi fold belt (Figure 10).

We validated the WSE behavior for the Bouguer gravity anomaly dataset of the Delhi fold belt region (Figure 10), which has been used in past studies [3,41,42]. The gravity anomaly of the study area (longitude 74° to 78° E and latitude 26° to 30° N) was derived from the gravity map series of India-2006 [43], which is a compilation of the datasets acquired by various organizations and has an average spacing of ~5 km. The region covers different geological formation boundaries of complex tectonic settings. We estimated the wavelet detail coefficients and calculated the MWE, TWE, and RWE of the coefficients to derive WSE. WSE demarcated the edges due to the different subsurface geological boundaries acting as sources (Figure 11). We observed major signatures corresponding to the Great Boundary Fault, the Delwara lineament, the Kaliguman lineament, and the boundary between the Malani igneous suite and DFB. The identified boundaries (marked as dashed lines: red and black) corroborated the crustal density models [41]. The sharp changes in WSE correlated with the boundaries reported by [3,42] and are marked as red lines (B1, L1, L2, L3, L4, B6, D1, and D2). The dashed black lines in the WSE plot show newly identified boundaries with sudden entropy contrast along the NW and northern directions. These variations were not interpreted and need validation in future work. The pattern of the source edges corroborated the previous results [3,41,42] with geological inferences that (i) the segment of DSR constituents are a manifestation of the flexural bulging of the Himalayan foreland basin and (ii) the boundaries indicate NW deflection trends, which support the occurrence of a NW deflection of the NE-trending deep domal underplated material at the Moho.

## 6. Conclusions

We studied the behavior of the informational property of wavelet space entropy for the identification of source edge boundaries of potential field data. The WSE method was effective for complex source geometries of prismatic bodies. Our performance analysis suggests that the WSE is a reliable edge detector that exhibited clear signatures of shallow and deep underground sources. When compared to the ASA, TDR, and THETA approaches, WSE and THDR performed better. The method had obvious limitations when dealing with severely noisy data; nonetheless, it performed well when Gaussian noise was added. We investigated the method for the two realistic cases of the Bishop model and the Delhi fold belt region to identify the boundaries of the sources. The method identified the edges of synthetic complex prismatic models (M1 and M2) and the NS-striking fault structures of the Bishop model. It also precisely derived the major geological boundaries of DFB and the surrounding region. The NE to NW changes in the pattern were prominent and were speculated in previous studies [41,42] of the Himalayan collision in the Eocene. The WSE and THDR methods performed equally well and outperformed our performance analysis. Our findings can be considered pioneering in the context that no detailed study had been performed to investigate entropy behavior in potential field source characterization. Potential field source edges of varied natures were discernable, with sharp changes having relatively high values related to the disordered behavior at those locations. Our results demonstrated that WSE can provide insight into the source boundaries of potential field datasets and has implications in geophysical exploration problems addressing source characterization. Wavelet space entropy provides useful subsurface structural information that can contribute to valuable maps.

## Figures and Tables

**Table 1:** The source geometries of the synthetic cases of the magnetic model (M1).

Model	Prismatic Source	Length (m)	Width (m)	Extent (m)	Total Magnetization (A/m)	Susceptibility (SI)	Z Top (m)	Inclination of Prism (Degree)
M1	S1	2400	400	700	2.6	+0.05	300	0
	S2	400	2400	700	2.6	+0.05	400	45
	S3	400	2400	700	2.6	+0.05	300	−45
	S4	2400	2400	400	2.6	+0.05	400	0
	S5	1000	1000	200	2.6	+0.05	200	0
	S6	300	300	200	2.6	+0.05	100	0

**Table 2:** The synthetic case after changing the parameters and geometries of the prismatic source of the model (M2).

Model	Prismatic Source	Length (m)	Width (m)	Extent (m)	Total Magnetization (A/m)	Susceptibility (SI)	Z Top (m)	Inclination of Prism (degree)
M2	S1	2600	600	700	2.1	+0.04	220	0
	S2	200	2200	700	−1.8	−0.04	220	45
	S3	200	2200	700	2.1	+0.04	250	−45
	S4	2000	2000	400	−2.3	−0.05	320	30
	S5	800	800	200	2.6	+0.05	150	0
	S6	200	200	200	3.2	+0.06	100	0

## Data Availability

Bishop model dataset can be found at: https://wiki.seg.org/wiki/Bishop_Model (accessed on 20 December 2022). Gravity data is restricted as per the government policy.

## Appendix A. Multiresolution Analysis Using DWT

The DWT uses multiresolution analysis to represent a signal in terms of its spatial frequency components [32]. It represents a signal using the approximation $\left(V_{j}\right)$ and detail $\left(W_{j}\right)$ spaces with different levels (k = 1 to D) of resolution.

A sequence ${\left\{V_{j}\right\}}_{j\in Z}$ of the closed subspaces of Hilbert space $L^{2}\left(R\right)$ is a multiresolution approximation if the following properties exist [32]:

For each integer j, the functions are an orthonormal basis for each $\;V_{j}$. The wavelet spaces $W_{j}$ are introduced from the orthogonal complements of $V_{j}$ in $\;V_{j-1}(V_{j-1}=V_{j}⊕W_{j},\;\forall j\in Z$. One can construct wavelet such that the dilated and translated family is an orthogonal basis of $\;L^{2}\left(R\right)$.

In 2D wavelet transform, the scaling and wavelet function are two variable functions, denoted as ϕ(x,y)  and  ψ(x,y).

The scaled and wavelet basis functions expressions are given as [32]:

(A1)
$$\phi_{j_{0},p,q}\left(x,y\right)=2^{j/2}\phi \left(2^{j}x-p,\;2^{j}y-q\right),$$

(A2) $$\psi_{j_{0},p,q}^{i}\left(x,y\right)=2^{j/2}\psi^{i}\left(2^{j}x-p,\;2^{j}y-q\right),\;\;\;\;\;\;\;\;\;\;\;\;\;\;i=\left\{H,V,D\right\}$$

where $\left(x,y\right)\in R^{2}\;$ and $\left(p,q\right)\in Z^{2}$.

There is one scaling function and three wavelet functions for each level (k). If the wavelet function is separable, then these functions can be rewritten as:

(A3)
ϕ(x,y)=ϕ(x) ϕ(y),

(A4)
$$\psi^{H}\left(x,y\right)=\psi \left(x\right)\;\phi \left(y\right),$$

(A5)
$$\psi^{V}\left(x,y\right)=\phi \left(x\right)\;\psi \left(y\right),$$

(A6) $$\psi^{D}\left(x,y\right)=\psi \left(x\right)\;\psi \left(y\right),$$

The wavelet family ${\left\{\psi_{j,p,q}^{H},\;\psi_{j,p,q}^{V},\;\psi_{j,p,q}^{D}\right\}}_{p,q\in Z^{2}}$ is an orthonormal basis of $W_{j}^{2}$ and ${\left\{\psi_{j,p,q}^{H},\;\psi_{j,p,q}^{V},\;\psi_{j,p,q}^{D}\right\}}_{\left(j,p,q\right)\in Z^{3}}$ is an orthonormal basis of $\;L^{2}\left(R^{2}\right)$.

The wavelet approximation coefficients for the function f(x,y)  of size M×N  can be given as [32]:

(A7) $$W_{\phi }\left(j_{0},p,q\right)=\frac{1}{\sqrt{MN}}\overset{M-1}{\underset{x=0}{\sum }}\overset{N-1}{\underset{y=0}{\sum }}f\left(x,y\right)\phi_{j_{0},p,q}\left(x,y\right)$$

where $\;j_{0}$ is the scale of the coefficients.

The horizontal $\;(W_{\psi }^{H})$, vertical $\left(W_{\psi }^{V}\right)$ and diagonal $\left(W_{\psi }^{D}\right)$ wavelet detail coefficients can be written as [32]:

(A8)
$$W_{\psi }^{H}\left(j,p,q\right)=\frac{1}{\sqrt{MN}}\overset{M-1}{\underset{x=0}{\sum }}\overset{N-1}{\underset{y=0}{\sum }}f\left(x,y\right)\psi_{j,p,q}^{H}\left(x,y\right)\;\;\;\;\;\;\;\;\;j\geq j_{0},$$

(A9)
$$W_{\psi }^{V}\left(j,p,q\right)=\frac{1}{\sqrt{MN}}\overset{M-1}{\underset{x=0}{\sum }}\overset{N-1}{\underset{y=0}{\sum }}f\left(x,y\right)\psi_{j,p,q}^{V}\left(x,y\right)\;\;\;\;\;\;\;\;\;j\geq j_{0},$$

(A10)
$$W_{\psi }^{D}\left(j,p,q\right)=\frac{1}{\sqrt{MN}}\overset{M-1}{\underset{x=0}{\sum }}\overset{N-1}{\underset{y=0}{\sum }}f\left(x,y\right)\psi_{j,p,q}^{D}\left(x,y\right)\;\;\;\;\;\;\;\;\;j\geq j_{0},$$

The wavelet transform (discrete or continuous) of a function is the improved version of the Fourier transform. The Fourier transform is a technique for analyzing the components of a stationary signal whereas the wavelet transform allows the components of a non-stationary signal to be analyzed. We have used the Daubechies wavelet of order 1 (db1) as the analyzing wavelet which comes in the family of orthogonal wavelets. These wavelets are characterized by a scaling function that generates an orthogonal multiresolution analysis.
